# Risk factors of post-stroke epilepsy among pediatric population: a systematic review

**DOI:** 10.3389/fneur.2025.1622551

**Published:** 2026-01-27

**Authors:** Saeed A. Alqahtani, Yamane A. Makke

**Affiliations:** 1 Department of Neurosurgery, MedStar Georgetown University Hospital, Washington, DC, United States; 2 Department of Neurology, Epilepsy Center, George Washington University Hospital, Washington, DC, United States

**Keywords:** epilepsy, pediatrics, risk, risk factor, stroke

## Abstract

Epilepsy is a frequent complication observed among stroke survivors. Post-stroke epilepsy (PSE) is defined as the occurrence of at least two unprovoked seizures beyond 2 weeks of an acute stroke that are not due to any other identifiable cause. PSE constitutes a significant clinical concern in pediatric stroke patients, adversely affecting both short-term and long-term management outcomes. This systematic review aimed to identify patient-related, stroke-related, and seizure-related risk factors associated with the development of PSE in the pediatric population. We included all clinical studies that compared these variables between pediatric stroke patients who developed PSE and those who did not. Studies reporting potential predictors of PSE among children with stroke were incorporated into the analysis. A total of 16 studies comprising 3,198 patients were included. The pooled risk of PSE was 27.6%, with a 95% confidence interval ranging from 19.8 to 37.2% (*p* < 0.001). Statistically significant associations were observed between younger age at stroke onset (OR 0.838; 95% CI 0.796–0.883; *p* < 0.001), cortical involvement (OR 3.151; 95% CI 1.132–8.772; *p* = 0.028), middle cerebral artery involvement (OR 3.541; 95% CI 1.068–11.738; *p* = 0.039), and increased risk of PSE. Additionally, patients presenting with acute symptomatic seizures (HR 3.924; 95% CI 2.580–5.967; *p* < 0.001) and those experiencing prolonged acute symptomatic seizures (OR 4.7; 95% CI 2.286–9.662; p < 0.001) demonstrated a higher likelihood of developing PSE. Pediatric patients who are younger at stroke onset and exhibit cortical or middle cerebral artery involvement are at a substantially elevated risk for PSE. Furthermore, the presence of acute symptomatic seizures at stroke onset, particularly when prolonged, markedly increases the probability of subsequent PSE development.

## Introduction

Stroke is a debilitating neurological injury associated with significant morbidity and mortality. The prevalence of stroke is more pronounced among the adult population due to the cumulative impact of comorbidities. Pediatric stroke is an acute neurological deficit in children aged 18 years or younger, with radiological evidence of cerebral infarction corresponding to the clinical manifestations (1). The death rate among children with stroke ranges from 10 to 25%, ranking among the top 10 causes of death in children (2–4). Pediatric stroke is classified into two types, hemorrhagic and ischemic, with an incidence of approximately 1.2 to 13 cases per 100,000 children. Pediatric stroke often results in death or considerable disabilities imposing serious consequences (5, 6). Despite advances in stroke management, the diagnosis of stroke among the pediatric population is usually delayed. Furthermore, there is a lack of knowledge regarding the most effective interventions for pediatric stroke. Therefore, the majority of pediatric stroke survivors experience long-term consequences with a greater loss of disability-free life years compared to adults. Notably, 50–85% of pediatric stroke survivors usually experience considerable behavioral problems, language impairment, sensorimotor deficits, and epilepsy (7, 8). The burden of pediatric strokes on the patients and healthcare systems warrants greater attention and further evaluation.

Epilepsy is a common complication among stroke survivors. Post-stroke epilepsy (PSE) is defined as the occurrence of at least two unprovoked seizures beyond 2 weeks of an acute stroke that are not due to any other identifiable cause. The incidence of PSE ranges from 4 to 13% among patients with hemorrhagic stroke and from 3 to 7% among patients with ischemic stroke (9–11). The 10-year cumulative incidence of PSE is nearly 30% among childhood stroke survivors (12). Importantly, PSE is attributable to the structural changes associated with stroke. This included neuronal loss, subsequent gliosis, and the significant remodeling of the neuronal networks (13, 14). In contrast, early or acute symptomatic seizures that occur within a week after stroke are attributed to metabolic dysfunction, abrupt disruption of cerebral integrity, and transient neuro-depolarization (15). PSE represents a significant threat in children with stroke and negatively impacts short- and long-term management. Pediatric patients have worse parent-reported health outcomes with substantial cognitive and performance disabilities (16). This included retardation in achieving independence, social responsibility, and learning disabilities (17).

Pediatric stroke is a rare entity that is often diagnosed with significant delays or misdiagnosed. PSE is associated with a high risk of all-cause mortality and morbidity. With improved survival after stroke, the impact of PSE on the quality of life is a growing area in the literature. Identifying risk factors of PSE is of great importance to improve the management of pediatric patients with stroke (9, 18). Several risk factors for PSE have been investigated among stroke survivors. These include stroke severity, lesion size, age at onset, cortical involvement, stroke subtype, and the presence of early seizures. Paradoxically, the findings of previous reports are often contradictory, with most articles focusing on adult populations. In this respect, substantial heterogeneity exists among these studies in terms of recruitment criteria, sampling methods, follow-up protocols, and the characteristics of the population (19–21). Consequently, the present systematic review aimed to identify patient-related, stroke-related, and seizure-related risk factors for epilepsy in the pediatric population following stroke. Understanding these risk factors is essential for improving prognostication and optimizing treatment strategies, thereby enhancing the quality of life for affected children and their families.

## Methods

This systematic review was conducted in accordance with the Preferred Reporting Items for Systematic Reviews and Meta-Analyses (PRISMA) guidelines (22), and the methodological recommendations of the Cochrane Collaboration (23). The study protocol was prospectively registered in the International Prospective Register of Systematic Reviews (PROSPERO) under the registration number CRD42023431824^1^.

### Data source

A comprehensive systematic literature search was conducted up to 30 May 2023 across the following databases: PubMed, NYAM, Google Scholar, EMBASE, ISI, SIGLE, Scopus, VHL, ClinicalTrials.gov, Controlled Trials (RCT), and the WHO International Clinical Trials Registry Platform (ICTRP). No restrictions were applied regarding patients’ sex, ethnicity, language, publication date, race, or geographical location. The search strategy employed various combinations of the following keywords: *Pediatric*, *Children*, *Neonate*, *Newborn*, *Infant*, *Neonatal*, *Adolescence*, *Stroke*, *Epilepsy*, and *Seizure*. An updated search was executed on 28 April 2025 to ensure the inclusion of the most recent studies. A manual search was also performed to recognize potentially relevant articles that were not indexed in electronic databases. Cross-referencing and citation tracking methods were also employed until no further eligible studies were included.

### Eligibility criteria

All clinical studies that compared patient-related, stroke-related, and seizure-related variables between pediatric stroke patients who developed PSE and those who did not were eligible for inclusion. Studies that investigated potential predictors of PSE in the pediatric stroke population were also included. No restrictions were applied regarding patients’ age, sex, race, or geographic location. However, studies involving pediatric patients with a prior history of epilepsy were excluded. Non-comparative studies, as well as those that did not report potential predictors of PSE, were also excluded. In addition, studies from which data could not be extracted, along with guidelines, review articles, animal studies, case reports, commentaries, letters to the editor, editorials, posters, and book chapters, were excluded from the analysis.

All identified articles were exported into an Excel spreadsheet. Titles, abstracts, and full-texts were screened independently to identify studies that fulfilled the inclusion criteria.

### Study definitions

- Acute symptomatic seizure: seizures occurring within 7 days of stroke onset.
- Prolonged symptomatic seizure: seizures occurring beyond 7 days after stroke onset.
- Post-stroke epilepsy: the occurrence of at least two unprovoked seizures beyond 2 weeks of an acute stroke that are not due to any other identifiable cause.
- Early seizures: occurring within 7–14 days of stroke onset.
- Neonatal Stroke: Cerebrovascular event occurring in newborns ≤28 days old.
- Pediatric Stroke: Cerebrovascular event occurring in children 1 month to 18 years old.
- Age at Stroke Onset: The child’s age when the stroke actually occurs (the time of the cerebrovascular event). This reflects the biological timing of the insult and is used to assess risk factors associated with stroke susceptibility and outcomes.
- Age at Stroke Diagnosis: The child’s age when the stroke is clinically recognized and confirmed (by imaging or clinical evaluation). This may be later than onset due to delayed recognition, especially in neonates or infants, and can affect treatment timing and prognosis.

### Data extraction and quality assessment

The following data were retrieved from the eligible articles; study characteristics (the title of the included study, the second name of the first author, year of publication, study design, study period, and study region), patient demographic characteristics (sample size, age, gender, race, and family history of epilepsy), stroke-related data (type of stroke, site of infarction, laterality, extent, cortical involvement, neurological deficit, early seizure, type of seizures, electroencephalography (EEG), and diagnostic criteria of PSE) and epilepsy-related data (time to epilepsy onset, Epilepsy-related characteristics, and neurological deficit). The quality of the observational studies will be assessed using the National Institute of Health (NIH) quality assessment tool (24).

### Statistical analysis

The risk of post-stroke epilepsy (PSE) was estimated by calculating the event rate and corresponding 95% confidence intervals (CIs) for each included study, followed by pooling these effect sizes to derive an overall summary proportion with a 95% CI. For dichotomous variables, risk ratios (RRs) or odds ratios (ORs) with 95% CIs were utilized. Additionally, pooled hazard ratios (HRs) or ORs were calculated by aggregating effect sizes from all eligible studies. A fixed-effect model was employed when a common effect size across studies was assumed; otherwise, a random-effects model was applied. Statistical heterogeneity was assessed using Higgins’ *I*^2^ statistic, with values exceeding 50% indicating substantial heterogeneity, and the Cochrane *Q* test (Chi^2^), with a significance threshold of *p* < 0.10 (24). In cases of notable heterogeneity, the random-effects model was used to account for variability among studies. Potential publication bias was evaluated through visual inspection of funnel plot asymmetry and Egger’s regression test, with *p* < 0.10 indicating possible bias (25). Data analysis was performed using Review Manager version 5.4 and Comprehensive Meta-Analysis (CMA) version 3 software (26, 27). A *p*-value < 0.05 was considered statistically significant.

## Results

**Figure 1:**
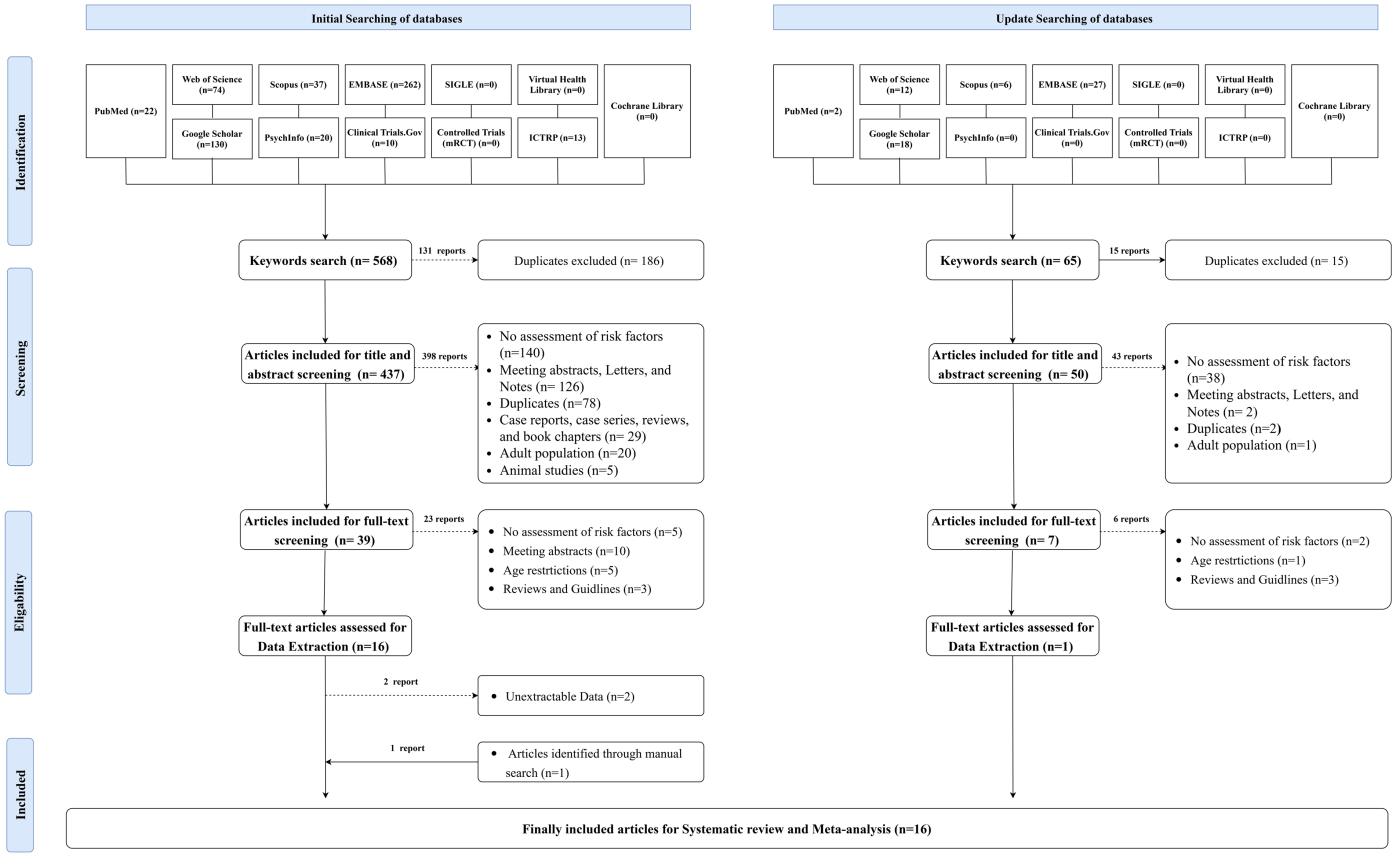
PRISMA flow chart showing the process of the literature search, title, abstract, and full text screening, systematic review, and meta-analysis.

A comprehensive search across 12 databases identified 568 articles. Following the removal of 131 duplicates, 437 studies remained for title and abstract screening. Of these, 398 reports were excluded, leaving 39 studies eligible for full-text screening. Subsequently, 23 articles were excluded, resulting in 16 studies eligible for data extraction, of which two were further excluded. An additional article was identified through citation tracking, culminating in 15 articles included in the initial meta-analysis. An updated database search later identified one more article, resulting in 16 articles finally being included for meta-analysis (Figure 1).

### Demographic characteristics of the included studies

The present systematic review and meta-analysis included 16 articles, encompassing 3,198 patients (12, 28–42). Eight of these studies employed a prospective design, while the remaining eight were retrospective. Five studies recruited patients from the United States, and two from Turkey. The mean age of patients with PSE ranged from 1 to 3.6 years, whereas among patients without PSE, the range was 1.5 to 9 years. The cohort included 1,205 males and 991 females. Ischemic stroke was diagnosed in 422 patients, while 214 patients experienced hemorrhagic stroke. Anterior circulation infarction was documented in 243 patients, compared to 64 patients with posterior circulation involvement (Table 1).

**Table 1:** Demographic characteristics of the included studies.

Study ID		Study region	Study design	Study period	Sample size		Age (years)		Gender				Type of stroke				Infarction location			
									Males		Females		Ischemic		Hemorrhagic		Anterior circulation		Posterior circulation	
					Epilepsy	No-epilepsy	Epilepsy	No-epilepsy	Epilepsy	No-epilepsy	Epilepsy	No-epilepsy	Epilepsy	No-epilepsy	Epilepsy	No-epilepsy	Epilepsy	No-epilepsy	Epilepsy	No-epilepsy
					Number	Number	Mean ± SD	Mean ± SD	Number	Number	Number	Number	Number	Number	Number	Number	Number	Number	Number	Number
1	Billinghurst et al. (2017) (28)	USA	Retrospective	January 1, 2006, and October 31, 2014	32	218	NR	NR	140		78		218		0		154		45	
2	Breitweg et al. (2016) (29)	Germany	Retrospective	1986 to 2003	22	72	40.5 months	108 months	15	36	10	32	12	36	13	32	NR	NR	NR	NR
3	Chadehumbe et al. (2009) (30)	USA	Prospective	July 1, 1993 to 1999	18	31	NR	NR	7	9	11	22	NR		NR		NR	NR	NR	NR
4	Fox et al. (2013) (12)	USA	Retrospective	January 1993– December 2007	40	305	NR	NR	NR	NR	NR	NR	140		165		NR	NR	NR	NR
5	Fox et al. (2016) (31)	USA	Retrospective	1993–2007	24	87	NR	NR	49		38		NR	NR	NR	NR	1		6	
6	Fox et al., 2017 (32)	Multicenter	Prospective	March 2011–August 2012	11	109	NR	NR	66		48		NR	NR	NR	NR	NR	NR	NR	NR
7	Hsu et al. (2014) (33)	Taiwan	Prospective	NR	17	78	3.6 ± 5.6	8.7 ± 5.9	35		38		NR	NR	NR	NR	NR	NR	NR	NR
8	Incecik et al. (2017) (34)	Turkey	Retrospective	January 2004 and January 2014	17	39	NR	NR	6	12	11	10	NR	NR	NR	NR	NR	NR	NR	NR
9	Kopyta et al. (2015) (35)	Poland	Prospective	2002 and 2012	7	65	2:9 ± 2:4	9 ± 5.5	4	35	3	23	NR	NR	NR	NR	5	36	0	7
10	Lopez-Espejo et al. (2024) (42)	Chile	Retrospective	2003 to 2019	68	48	NR	NR	37	NR	31	NR					47		6	
11	Lvova et al. (2016) (37)	Russia	Prospective	NR	22	136	NR	NR	NR	NR	NR	NR	NR	NR	NR	NR	NR	NR	NR	NR
12	López-Espejo et al. (2018) (36)	Chile	Prospective	January 2003 and July 2013	40	72	NR	NR	60		38		NR	NR	NR	NR	NR	NR	NR	NR
13	Mineyko et al. (2020) (38)	Canada and USA	Prospective	NR	6	24	1.0(0.4–12.8)*	1.5(0.1–4.6)*	3	13	1	4	2	14	2	2	NR	NR	NR	NR
14	Polat et al. (2021) (39)	Turkey	Retrospective	2012 and 2016	36	51	3.1 ± 4.6	2.8 ± 4.0	24	11	12	4	NR	NR	NR	NR	NR	NR	NR	NR
15	Sundelin et al. (2021) (40)	Sweden	Prospective	1969 to 2016	219	1,220	NR	NR	643		577		NR	NR	NR	NR	NR	NR	NR	NR
16	Suppiej et al. (2016) (41)	Italy	Retrospective	January 1990 to 30th June 2010	9	55	NR	NR	NR	NR	NR	NR	NR	NR	NR	NR	NR	NR	NR	NR

SD, standard deviation, *Data reported using median and range.

Early seizures were observed in 222 patients with PSE and 37 patients without PSE. Among those with PSE, 14 experienced generalized seizures, and 15 had focal seizures. Cortical involvement was reported in 99 patients with PSE and 38 patients without PSE. The mean follow-up duration ranged from 12.3 months to 8.7 years. The quality of the included observational articles was assessed as good according to NIH quality assessment tool (Table 2).

**Table 2:** Characteristics of patients with post-stroke epilepsy and quality assessment.

Study ID		Early seizures		Types of seizures				EEG findings				Follow-up period	Quality assessment	
				Generalized		Focal		Cortical Involvement		Neurological Deficit				
		Epilepsy	No-epilepsy	Epilepsy	No-epilepsy	Epilepsy	No-epilepsy	Epilepsy	No-epilepsy	Epilepsy	No-epilepsy			
		Number	Number	Number	Number	Number	Number	Number	Number	Number	Number		%	Decision
1	Billinghurst et al. (2017) (28)	NR	NR	NR	NR	NR	NR	NR	NR	NR	NR	34 (16.3–61.2) Months	75%	Good
2	Breitweg et al. (2016) (29)	15	4	NR	NR	NR	NR	8	16	NR	NR	2 Years	75%	Good
3	Chadehumbe et al. (2009) (30)	NR	NR	NR	NR	NR	NR	NR	NR	NR	NR	4.1 years (IQR 1.8–6.8)	66.66%	Good
4	Fox et al. (2013) (12)	NR	NR	NR	NR	NR	NR	NR	NR	NR	NR	7.1 (3.2–10)	75%	Good
5	Fox et al. (2016) (31)	58	NR	NR	NR	NR	NR	NR	NR	NR	NR	12.3 months (IQR 11.6–13)	75%	Good
6	Fox et al. (2017) (32)	NR	NR	NR	NR	NR	NR	NR	NR	NR	NR	53.7 ± 48.8 months (range, 1–191 months)	66.66%	Good
7	Hsu et al. (2014) (33)	7	19	4	8	13	13	13	10	15	22	2 years.	66.66%	Good
8	Incecik et al. (2017) (34)	NR	NR	NR	NR	NR	NR	NR	NR	NR	NR	NR	66.66%	Good
9	Kopyta et al. (2015) (35)	NR	NR	10	18	2	9	NR	NR	NR	NR	NR	75%	Good
10	Lvova et al. (2016) (37)	52	NR	NR	NR	NR	NR	NR	NR	NR	NR	3 years	66.66%	Good
11	López-Espejo et al. (2018) (36)	NR	NR	NR	NR	NR	NR	NR	NR	NR	NR	NR	75%	Good
12	Lopez-Espejo et al. (2024) (42)	55	NR	NR	NR	NR	NR	50	NR	NR	NR	23.7 ± 14.8 Months	75%	Good
13	Mineyko et al. (2020) (38)	2	7	NR	NR	NR	NR	NR	NR	NR	NR	NR	66.66%	Good
14	Polat et al. (2021) (39)	33	7	NR	NR	NR	NR	28	12	27	10	NR	75%	Good
15	Sundelin et al. (2021) (40)	NR	NR	NR	NR	NR	NR	NR	NR	NR	NR	8.7 years	75%	Good
16	Suppiej et al. (2016) (41)	NR	NR	NR	NR	NR	NR	NR	NR	NR	NR	8 years	66.66%	Good

IQR, interquartile range.

### The risk of post-stroke epilepsy

**Figure 2:**
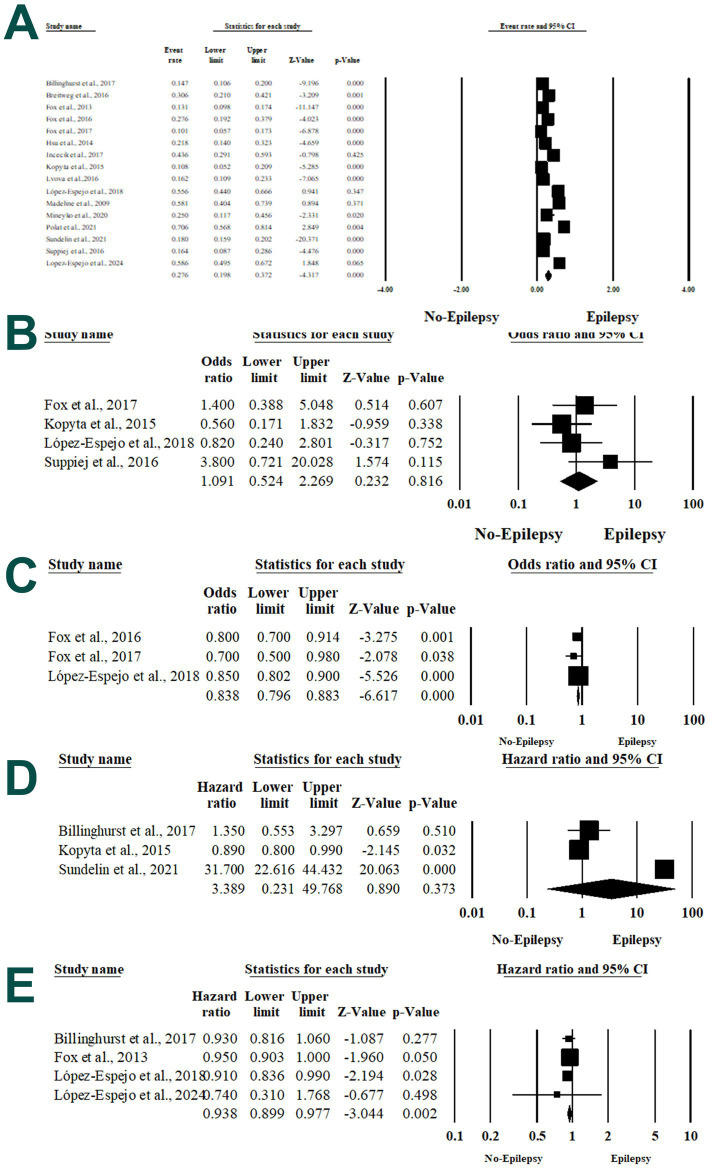
Forest plot of summary analysis of the **(A)** event rate and 95% CI of the risk of post-stroke epilepsy. **(B)** Odds ratio and 95% CI of the association between male gender and post-stroke epilepsy. **(C)** Hazard ratio and 95% CI of the association between the age at stroke onset and post-stroke epilepsy. **(D)** Hazard ratio and 95% CI of the association between the age at stroke diagnosis and post-stroke epilepsy. **(E)** Hazard ratio and 95% CI of the association between younger age at stroke onset and post-stroke epilepsy. Size of the black squares is proportional to the statistical weight of each trial. The gray diamond represents the pooled point estimate. The positioning of both diamonds and squares (along with 95% CIs) beyond the vertical line (unit value) suggests a significant outcome (IV, inverse variance).

Sixteen articles included 3,198 patients with stroke (12, 28–42). Of them, 588 patients developed PSE. Pooling the data in the random-effects model (*I*^2^ = 93.7%, *p* < 0.001), revealed a risk of PSE of 27.6%, ranging from 19.8 to 37.2% (*p* < 0.001). There was no evidence of publication bias based on the symmetrical distribution of studies along the middle line of the funnel plot and based on Egger’s regression test (Intercept = 2.53, *p* = 0.14) (Figure 2A).

## Factors associated with post-stroke epilepsy

### Patient-related factors

#### Male gender

Four studies (32, 35, 36, 41)reported the association between male gender and PSE. There was no significant association between both variables (OR 1.091; 95% 0.524, 2.269; *p* = 0.816) in the random-effects model (*I*^2^ = 19.5%, *p* = 0.292) (Figure 2B).

#### Age at stroke onset

Three articles reported the association between the age at stroke onset and the risk of PSE (28, 35, 40). There was a statistically significant association between the age at stroke onset and the risk of PSE with an odds ratio of 0.838 and 95%CI ranging from 0.796 to 0.883 (*p* < 0.001), in the random-effects model (*I*^2^ = 0%, *p* = 0.4) (Figure 2C).

### Stroke-related factors

#### Age at stroke diagnosis

Three articles evaluated the association between age at stroke diagnosis and the risk of PSE (12, 28, 36). In the random-effects model (*I*^2^ = 0%, *p* = 0.687), there was no increase in the risk of PSE with age at stroke diagnosis (HR 3.389; 95% 0.231, 49.768; *p* = 0.816) (Figure 2D).

#### Younger age at stroke onset

Four studies assessed the association between younger age at stroke onset and PSE (12, 28, 36, 42). There was a statistically significant association between the two variables in the random-effects model (*I*^2^ = 79%, *p* < 0.001), with an HR of 0.938 (95% 0.899, 0.977; *p* = 0.002) (Figure 2E).

#### Neonatal arterial stroke

**Figure 3:**
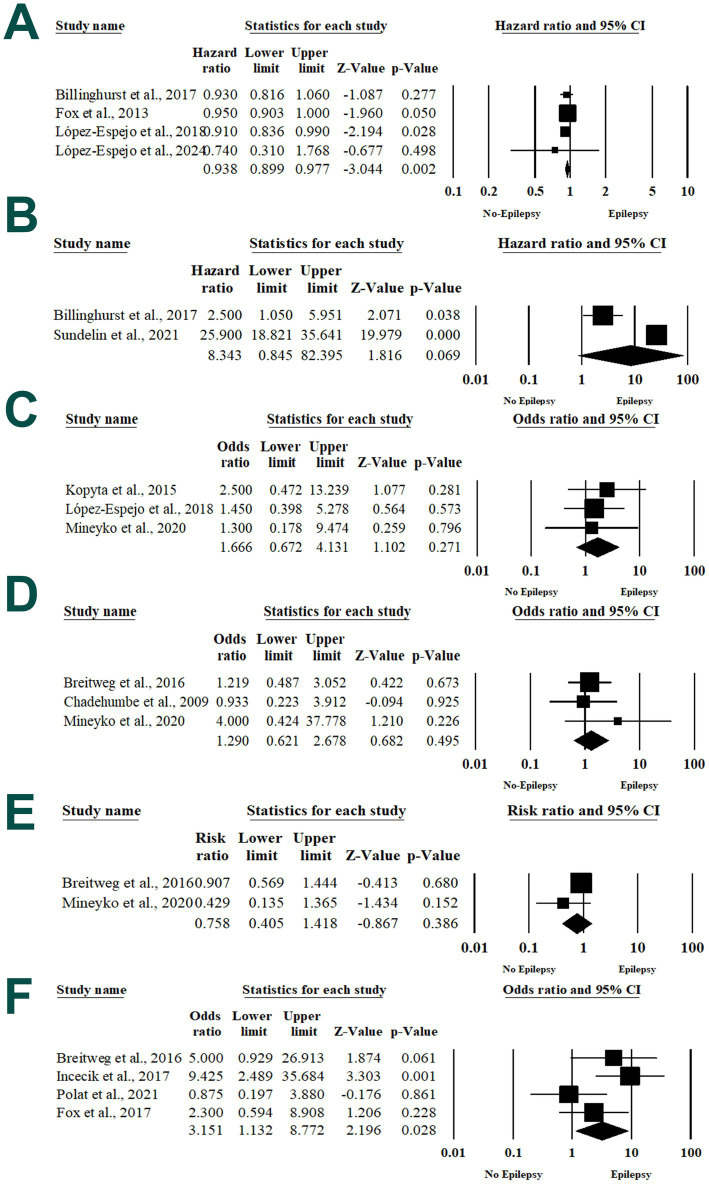
Forest plot of summary analysis of the **(A)** hazard ratio and 95% CI of the association between neonatal arterial stroke and post-stroke epilepsy. **(B)** Hazard ratio and 95% CI of the association between presumed perinatal arterial ischemic stroke and post-stroke epilepsy. **(C)** Odds ratio and 95% CI of the association between acute infection and post-stroke epilepsy. **(D)** The risk ratio and 95% CI of the association between hemorrhagic stroke and post-stroke epilepsy. **(E)** The risk ratio and 95% CI of the association between ischemic stroke and post-stroke epilepsy. **(F)** Odds ratio and 95% CI of the association between cortical involvement and post-stroke epilepsy. Size of the black or blue squares is proportional to the statistical weight of each trial. The gray diamond represents the pooled point estimate. The positioning of both diamonds and squares (along with 95% CIs) beyond the vertical line (unit value) suggests a significant outcome (IV, inverse variance).

The association between neonatal arterial strokes and the risk of PSE was evaluated within three articles (28, 40, 42). Pooling the effect sizes (HR) in the random-effects model (*I*^2^ = 98.68%, *p* < 0.001) revealed no statistically significant association between the two variables (HR 3.311; 95% 0.254, 43.173; *p* = 0.360) (Figure 3A).

#### Presumed perinatal arterial ischemic stroke

The risk of PSE among patients with presumed perinatal arterial ischemic stroke was revealed within two studies (28, 40). There was no statistically significant association between the presumed perinatal arterial ischemic stroke and PSE with an HR of 8.343 (95% 0.845, 82.395; *p* = 0.069) in the random-effects model (*I*^2^ = 95.9%, *p* < 0.001) (Figure 3B).

#### Acute infection

Three articles reported the association between acute infection and PSE (35, 36, 38). In the random-effects model (*I*^2^ = 0%, *p* = 0.847), there was no increase in the risk of PSE with acute infection at the stroke onset (OR 1.66; 95% 0.672, 1.102; *p* = 0.271) (Figure 3C).

#### Types of stroke

Three articles reported the risk of PSE after hemorrhagic stroke (29, 30, 38). There was no statistically significant higher risk of PSE among patients with hemorrhagic stroke (*p* = 0.553) with a RR of 1.123 and 95% CI ranging from 0.766 to 1.646, in the random-effects model (*I*^2^ = 0%, *p* = 0.498). Two articles evaluated the risk of PSE after ischemic stroke (29, 38). There was no statistically significant impact of ischemic stroke on the risk of PSE (RR 0.758; 95% 0.405, 1.418; *p* = 0.386) in the random-effects model (*I*^2^ = 27.7%, *p* = 0.23) (Figures 3D,E).

#### Cortical involvement

Four articles evaluated the association between cortical involvement and the risk of PSE (29, 32, 34, 39). Patients with cortical involvement were 3.151 times more vulnerable to developing PSE, in contrast to patients with cortical involvement (OR 3.151; 95% 1.132, 8.772; *p* = 0.028) in the random-effects model (*I*^2^ = 49.49%, *p* = 0.11) (Figure 3F).

#### Central nervous system (CNS) arteriopathy

**Figure 4:**
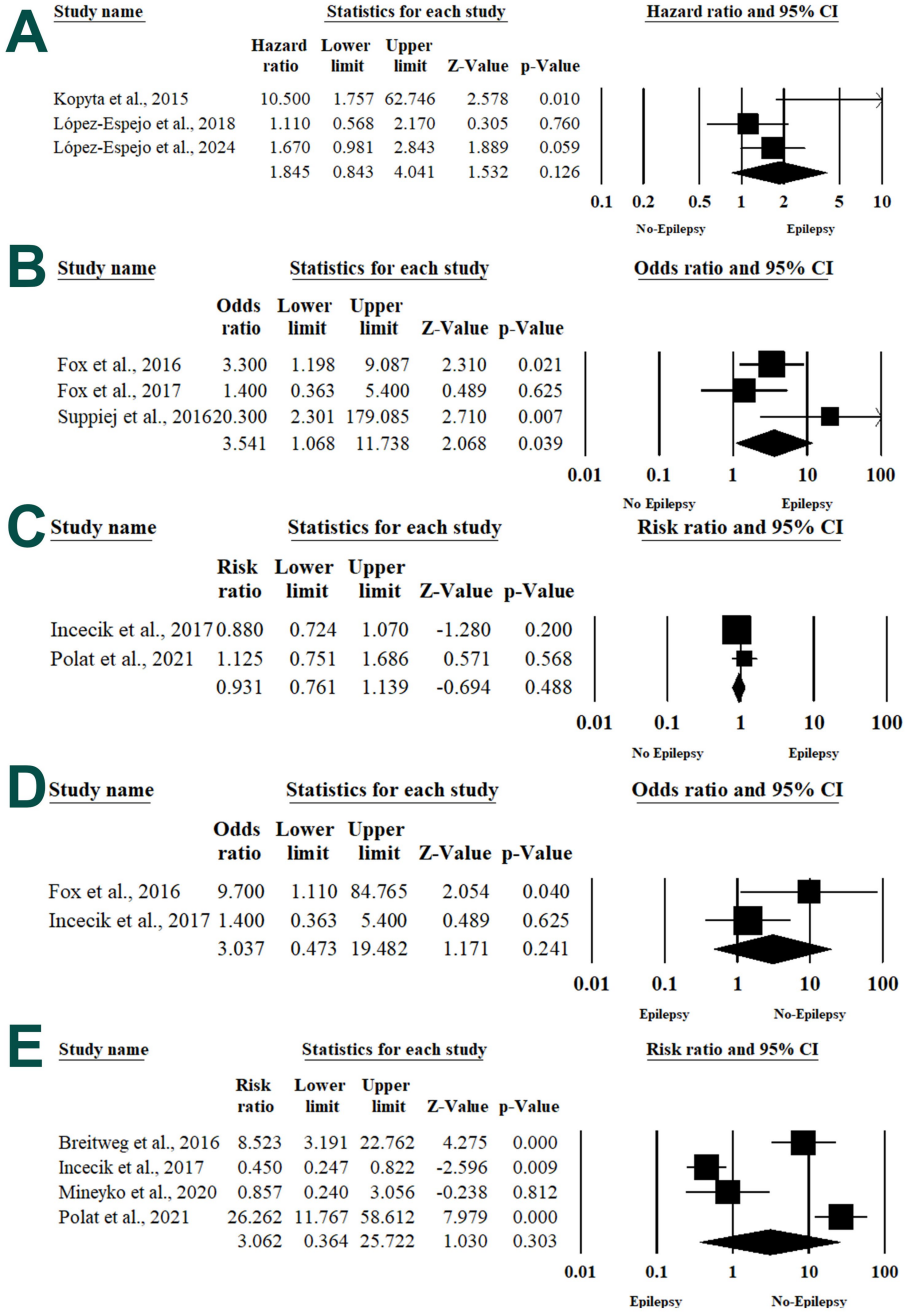
Forest plot of summary analysis of the **(A)** hazard ratio and 95% CI of the association between central nervous system arteriopathy and post-stroke epilepsy. **(B)** Odds ratio and 95% CI of the association between middle cerebral artery involvement and post-stroke epilepsy. **(C)** The risk ratio and 95% CI of the association between neurological deficit and post-stroke epilepsy. **(D)** The odds ratio and 95% CI of the association between abnormal EEG findings and post-stroke epilepsy. **(E)** the risk ratio and 95% CI of the association between early seizures and post-stroke epilepsy. Size of the black or blue squares is proportional to the statistical weight of each trial. The gray diamond represents the pooled point estimate. The positioning of both diamonds and squares (along with 95% CIs) beyond the vertical line (unit value) suggests a significant outcome (IV, inverse variance).

The association between CNS arteriopathy and PSE risk was evaluated in three articles (35, 36, 42). There was no statistically significant higher risk of PSE with CNS arteriopathy with an HR of 1.845 and 95% CI ranged from 0.843 to 4.041 with a *P*-value of 0.126 with significant heterogeneity between the included studies (*I*^2^ = 63.09%, *p* = 0.067) (Figure 4A).

#### Middle cerebral artery involvement

Three articles determined the risk of PSE among patients with middle cerebral artery involvement (31, 32, 41). Pooling the data in the random-effects model (*I*^2^ = 49.49%, *p* = 0.11) revealed a 3.541 times higher risk of PSE among patients with middle cerebral artery involvement (OR 3.541; 95% 1.068, 11.738; *p* = 0.039) (Figure 4B).

#### Neurological deficit

The risk of PSE associated with neurological deficit at the time of stroke was assessed within two articles (34, 39). Meta-analysis revealed no statistically significant association between neurological deficit and PSE (RR 0.931; 95% 0.761, 1.139; *p* = 0.488) with substantial heterogeneity between the included studies (*I*^2^ = 12.7%, *p* = 0.28) (Figure 4C).

#### Abnormal EEG findings

Two articles evaluated the association between abnormal EEG findings and the risk of PSE (31, 34). In the random-effects model (*I*^2^ = 54.6%, *p* = 0.13), there was no increase in the risk of PSE with acute infection at the stroke time (OR 3.037; 95% 0.473, 19.482; *p* = 0.241) (Figure 4D).

### Seizure-related variables

#### Timing of seizures

The risk of PSE among patients with early seizures was evaluated within four articles (29, 34, 38, 39). No statistically significant association was identified between early seizures and post-stroke epilepsy (PSE), with a relative risk (RR) of 3.062 and a 95% confidence interval (CI) ranging from 0.364 to 25.722 (*p* = 0.303). Notably, significant heterogeneity was observed among the included studies (*I*^2^ = 95.8%, *p* < 0.001) (Figure 4E).

**Figure 5:**
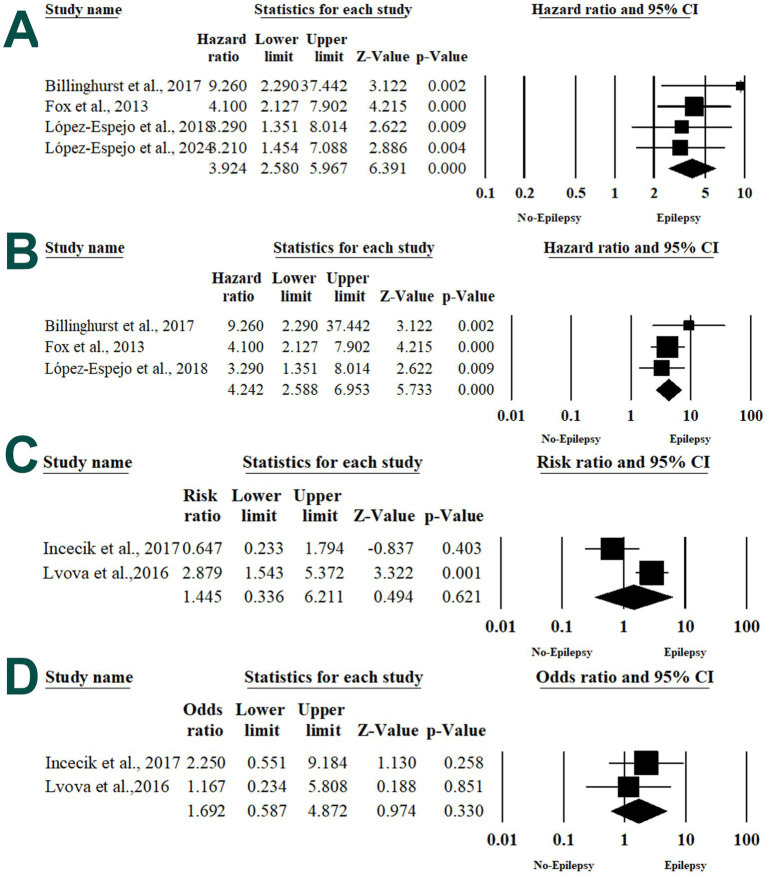
Forest plot of summary analysis of the **(A)** the risk ratio and 95% CI of the association between acute symptomatic seizures and post-stroke epilepsy. **(B)** Odds ratio and 95% CI of the association between duration of acute symptomatic seizures and post-stroke epilepsy. **(C)** The risk ratio and 95% CI of the association between generalized seizures and post-stroke epilepsy. **(D)** The risk ratio and 95% CI of the association between focal seizures and post-stroke epilepsy. Size of the black or blue squares is proportional to the statistical weight of each trial. The gray diamond represents the pooled point estimate. The positioning of both diamonds and squares (along with 95% CIs) beyond the vertical line (unit value) suggests a significant outcome (IV = inverse variance).

Four articles examined the association between acute symptomatic seizures and PSE (12, 28, 36, 42). Patients with acute symptomatic seizures were at 3.924 times higher risk of developing PSE (HR 3.924; 95% 2.580, 5.967; *p* < 0.001) without significant heterogeneity between the included studies (*I*^2^ = 0%, *p* = 0.6) (Figure 5A).

#### Duration of acute seizures

The association between the duration of acute symptomatic seizures and PSE risk was evaluated in two articles (31, 32). Patients with prolonged duration of acute symptomatic seizures were 4.7 times more at higher risk of developing PSE (OR 4.7; 95% 2.286, 9.662; *p* < 0.001) without significant heterogeneity between the included studies (*I*^2^ = 0%, *p* = 1) (Figure 5B).

#### Type of seizures

Two articles evaluated the risk of PSE among patients with generalized or focal seizures at the stroke presentation (34, 37). There was no statistically significant higher risk of PSE among patients with generalized seizures (RR 1.445; 95% 0.336, 6.211; *p* = 0.621) and focal seizures (RR 1.692; 95% 0.587, 4.872; *p* = 0.330) (Figures 5C,D).

## Discussion

The development of PSE is a debilitating consequence of stroke. The incidence of PSE among the pediatric population remains inadequately identified, with various estimations. Subsequently, predictors of PSE among the pediatric population have not been comprehensively investigated, and there are contradictory findings from previously published studies (43). The management and prognostication of PSE remain a challenge in the literature (21). The present systematic review highlighted the significant risk of PSE among pediatric stroke survivors. This risk was determined in approximately one of every three patients after a stroke. Patients younger at stroke onset, those with cortical involvement, or those with middle cerebral artery involvement at the time of stroke were at higher risk of PSE. Subsequently, patients with acute or prolonged seizures were at higher risk of PSE. Neither the type of stroke nor the presence of neurological deficits was associated with a significantly higher risk of PSE. These findings highlighted the urgent need to establish guidelines for diagnosing and treating pediatric patients at higher risk of PSE early and effectively. This systematic review revealed the significant risk of PSE among pediatric patients with stroke. The higher risk of PSE reflects the severity of stroke, conferring a considerable risk of long-term morbidities and mortality (44). Rattani et al. (45) reported a risk of PSE of 27.2% among perinatal arterial ischemic stroke survivors with an average duration of approximately 10 years since stroke onset. In parallel with these findings, Ren et al. (18) reported a significant association between PSE and all-cause mortality, suggesting the independent impact of PSE on death after stroke. Phan et al. (21) reported an increased risk of PSE among the younger population, male gender, and stroke affecting the cortex.

The present systematic review confirmed the association between younger age at stroke onset and PSE. Patients with neonatal arterial or perinatal ischemic strokes were at approximately six- and eight-times higher risk of PSE. These findings are consistent with Gao et al. (1) study. They revealed the significant neurological impairment associated with neonatal ischemic stroke. The average time to diagnose neonates with ischemic stroke is 22 h from the beginning of symptoms. Such a delay in the diagnosis is associated with a delay in management beyond the time window for intravenous thrombolysis or thrombectomy. This is associated with life-threatening consequences, highlighting the utmost importance of early diagnosis and effective therapy for stroke patients (46).

Patients with acute or prolonged seizures at the onset of stroke were associated with a higher risk of PSE. Anti-epileptic medications in such patients can be clinically significant in decreasing the risk of PSE (47). Similarly, Rattani et al. (45) confirmed the association between seizures at the stroke onset and PSE among perinatal arterial ischemic stroke survivors. Pediatric patients with acute symptomatic seizures are more likely to have epileptiform discharges and electrographic seizures on EEG. Furthermore, Patients with cortical involvement or middle cerebral artery involvement were at higher risk of PSE. This finding was consistent with Zhang et al. (19) who reported a significant risk of late seizures among patients with cortical involvement. Such patients may benefit from close monitoring and prophylactic anti-convulsive medications. However, the present systematic review showed a non-significant association between abnormal EEG changes and the risk of PSE. This finding supports the phenomenon of electroclinical dissociation in which a considerable number of neonatal seizures are subclinical. This highlighted the need for closer electroencephalographic monitoring of the pediatric population after stroke (48). Abnormal acute EEG findings may not reliably predict post-stroke epilepsy in neonates due to electroclinical dissociation, where epileptiform activity may occur without overt clinical seizures. Immature cortical networks and evolving seizure semiology further limit EEG sensitivity, underscoring the need for cautious interpretation and long-term clinical follow-up. Further studies are needed to evaluate the impact of prophylactic anti-epileptic medications on the long-term risk of PSE among the pediatric population.

This systematic review synthesized existing evidence concerning the risk factors associated with PSE. While the study possesses several strengths, certain limitations warrant consideration. All included studies employed observational designs, with a substantial proportion being retrospective, thereby introducing a potential risk of information bias. Additionally, variability existed among the studies in terms of PSE definitions, follow-up durations, age ranges, and statistical methodologies. This heterogeneity was reflected in the meta-analysis through significant statistical variability across multiple outcomes. To address this, a random-effects model was applied. Further research is necessary to elucidate the long-term effects of stroke on the risk of PSE and its subsequent clinical outcomes.

## Conclusion

The present systematic review highlights the significant risk of PSE among pediatric stroke survivors. Younger pediatric patients, as well as those with cortical or middle cerebral artery involvement, demonstrate an increased susceptibility to developing PSE. Furthermore, the occurrence of acute symptomatic seizures at stroke onset, especially when prolonged, substantially elevates the risk of subsequent epilepsy. Early identification of pediatric patients at heightened risk for PSE may enable clinicians to target vulnerable populations and implement timely preventive interventions aimed at reducing the incidence of PSE.

## Data Availability

The data analyzed in this study is subject to the following licenses/restrictions: the datasets used in the present study are available from the first author and corresponding authors on reasonable request. Requests to access these datasets should be directed to saeedkanee@gmail.com.

## Footnotes

1
http://www.crd.york.ac.uk/prospero/
